# The effect of wastewater effluent derived ligands on copper and zinc complexation

**DOI:** 10.1007/s11356-016-8332-3

**Published:** 2017-02-07

**Authors:** C. Constantino, S. D. W. Comber, M. D. Scrimshaw

**Affiliations:** 1 Atkins Limited, Chilbrook, Oasis Business Park, Eynsham, Witney, OX29 4AH UK; 2 Biogeochemistry Research Centre, Plymouth University, Plymouth, PL4 8AA UK; 3 Institute of Environment, Health and Societies, Brunel University, Uxbridge, UB8 3PH UK

**Keywords:** Copper, Zinc, Metal speciation, Biotic ligand model, Complexation, Sewage effluent

## Abstract

The shift toward bioavailability-based standards for metals such as copper and zinc not only improves the ecological relevance of the standard but also introduces significant complexity into assessing compliance. This study examined differences in the copper and zinc complexation characteristics of effluents from a range of different sewage treatment works and in relation to so-called ‘natural’ samples. This information is essential to determine whether the inclusion of effluent-specific complexation characteristics within the regulatory framework could enhance the environmental relevance of compliance criteria. The data show that for copper, binding affinity was not greater than that measured for materials derived from the receiving water environment, with a mean log *K* of between 4.4 and 5.15 and mean complexation capacity ranging from 38 to 120 μg/mg dissolved organic carbon (DOC) for effluents compared with a log *K* of 5.6 and complexation capacity of 37 μg/mg DOC for the Suwannee River fulvic acid. For zinc, however, effluents exhibited a much higher complexation capacity, with effluent means ranging from 3 to 23 μg/mg DOC compared with the Suwannee River fulvic acid, for which the complexation capacity could not be determined. Synthetic ligands in sewage effluent, such as ethylenediaminetetraacetic acid (EDTA), are implicated as contributing to these observed differences. This suggests that the current biotic ligand models for zinc might overstate the risk of harm in effluent-impacted waters. The data also show that the copper and zinc complexation characteristics of effluent samples obtained from the same sewage treatment works were less different from one another than those of effluents from other treatment works and therefore that sewage source has an important influence on complexation characteristics. The findings from this study support the case that the contribution to complexation from effluent-derived ligands could enhance the environmental relevance of bioavailability-based compliance criteria, in particular for zinc, owing to the additional complexation capacity afforded by effluent-derived ligands.

## Introduction

The environmental regulator for England, the Environment Agency, has recently specified new water quality standards for the Specific Pollutants, copper and zinc, in terms of a permissible bioavailable concentration under the Water Framework Directive (WFD) (UKTAG 2012). The same directive has set bioavailability-based standards for the Priority Substances nickel and lead at the European Union level (EU 2000) and some states in America have established bioavailability-based values for copper USEPA (2007). The permissible bioavailable concentration represents the metal concentration that is in a form available to be taken up by aquatic organisms but at a level at which adverse ecotoxicological impacts are not expected to occur. The consideration of bioavailability not only improves the ecological relevance of water-quality standards but also introduces significant complexity into assessing compliance. The complexity arises since the bioavailable concentration cannot be measured directly and requires the use of a Biotic Ligand Model (BLM) to ‘convert’ a measured dissolved metal concentration into a bioavailable metal concentration equivalent (Di Toro et al. 2001) against which compliance may be assessed.

BLMs require, as a minimum, water chemistry data such as pH, and the concentrations of dissolved organic carbon (DOC) and calcium as inputs, where the effect of DOC on metal bioavailability is simulated on the assumption that DOC is comprised of humic substances which form metal complexes of a particular type (Di Toro et al. 2001; Santore et al. 2001). The organic molecules that DOC is comprised of are known to bind, or complex, metal ions and substantially reduce metal bioavailability (Playle et al. 1992, 1993; Al-Reasi et al. 2013). The extent by which BLMs might accurately reflect bioavailability in waters consisting substantially of sewage effluent is, however, uncertain since the current suite of BLMs has been parameterised using data from natural and reconstituted waters only.

Previous studies have shown that wastewater effluent ligands are capable of binding trace elements present in the matrix to a greater degree than naturally derived DOC from, for example, standard Suwannee River humic and fulvic acids (Chaminda et al. 2008, 2013) or that derived from road runoff (Chaminda et al. 2010). Furthermore, effluent-derived DOC has been shown to be different in form compared to DOC derived from natural sources. Studies which have investigated the chemical composition of sewage effluents (Ma et al. 2001; Pernet-coudrier et al. 2008) found approximately that 50% of the carbon content in sewage effluent-derived organic matter was comprised of an acid soluble hydrophilic fraction made up of high molecular weight (>3500 Da) polysaccharides, a value in excess of the proportion which occurs in naturally derived organic matter (between 9 and 30%). Sarathy and Allen (2005) also proposed that effluent-derived DOC should be included as an additional ligand type in biotic ligand modelling, and this has been supported by findings from other studies (Pernet-coudrier et al. 2008; Baken et al. 2011; Constantino et al. 2011).

Effluents are also known to contain non-humic anthropogenic substances which display metal complexing characteristics that differ from that of humics (Sarathy and Allen 2005). Indeed, effluents are complex mixtures containing multiple substances including compounds such as ethylenediaminetetraacetic acid (EDTA) (Knepper 2003; Knepper et al. 2005) and other ‘EDTA-like’ substances (Peters et al. 2014) that are known to bind metal ions that might influence metal bioavailability in an unpredictable manner, such that the extent to which the current suite of BLMs might accurately predict that metal toxicity is uncertain. Although for models, an agreement with measured data by within a factor of two is often considered sufficient, the presence of additional strong ligands at significant concentrations may easily lead to model estimates falling outside of this accepted variability.

A study by Baken et al. (2011), which investigated the metal complexing properties of organic matter in anthropogenically impacted surface waters, confirmed the significance of synthetic chelating agents such as EDTA to trace metal speciation, for nickel in particular. Recent data for 162 UK wastewater treatment works (WwTW) effluents have reported median EDTA concentrations of 128 μg/L (5.3 and 22.9 times greater than those of dissolved zinc and copper, respectively) (Gardner et al. 2012). However, whereas EDTA is arguably a well-known synthetic chelating agent, numerous other anthropogenic substances with chelating properties, such as organophosphonates, are also known, or may reasonably be expected, to occur in sewage effluents (Knepper 2003). In addition to occurring in their primary form, chelating agents might also occur as breakdown products; for example, ethylenediaminetriacetic acid (ED3A) has been identified as a common EDTA breakdown product (Nowack and VanBriesen 2005), which is also known to have a strong affinity for copper (log *K* 10.5) (Stumm and Morgan 1996).

The extent to which effluent-derived organic matter might affect the performance of BLMs is of particular importance since many parts of the UK (and elsewhere) have low effluent dilution capacity (≤1:10) (Keller et al. 2006) and river waters may therefore contain a substantial amount of effluent-derived organic matter. High proportions of effluent in river water may have implications for the accuracy of BLM estimates of metal bioavailability, and ultimately, the effectiveness of actions aimed at improving environmental conditions. For example, WwTWs can represent a significant source of copper and zinc inputs into the aquatic environment although most WwTWs are not designed to remove trace metals and consequently, removal is variable (Inna et al. 2014; Crane et al. 2010). Achieving metal removal rates in order to comply with bioavailability-based standards may require additional wastewater treatment processes that may be disproportionately expensive or energy (carbon) intensive. Whereas, improving the accuracy of biotic ligand models is, from a scientific perspective, valuable in itself, the prospect that BLMs will be applied within a compliance-based regulatory framework provides an additional imperative. Failure to take into account real-world effects may result in statutory standards which are either insufficiently protective so that harm may occur, or over-precautionary so that measures to reduce metal concentrations may be required, but which deliver no environmental benefit.

This study set out to assess differences in the copper and zinc complexation characteristics of effluents from different sewage treatment works, including the possible effect of (i) iron (Fe), which, not only being present in crude sewage from anthropogenic sources, is used extensively in wastewater treatment as a coagulant to control phosphorus in effluents, and (ii) synthetic chelating agents such as EDTA. Although the focus was predominantly on sewage effluents, to provide a comparison with ligands derived from river water sources, a standard fulvic acid (Suwannee River fulvic acid (SRFA)) was included in the analysis to compare the relative binding strengths and capacities of ligands from different sources. It is accepted that there are a variety of naturally occurring ligands present in river waters capable of complexing Cu and Zn; however, their inclusion within the experiments carried out here was both beyond the scope and practicalities of the research. There is, however, an abundance of literature already available for naturally occurring ligands. An understanding of the differences in the copper and zinc complexation characteristics of effluents from different sewage treatment works, and in relation to so-called natural ligands, is nonetheless required to determine whether the consideration of site-specific complexation characteristics within the regulatory framework could enhance the environmental relevance of compliance criteria wherever effluents are discharged to receiving waters in significant proportions.

## Materials and Methods

### Sample selection

Two effluent samples were obtained from each of four wastewater treatment works, designated A, B, C and D. All WwTWs utilised the activated sludge process (Metcalf and Eddy 2002) and received mostly domestic wastewater inputs. The activated sludge process treatment type is used widely and produces a relatively high quality effluent that is low in ammonia and chemical oxygen demand. Two treatment works (A and B) were known to receive inputs from dairies and were expected to contain high concentrations of EDTA as a consequence of its use in cleaning detergents that are used within the dairy industry (ECB 2004). Effluents from the other works (C and D) were expected to contain EDTA concentrations resulting from domestic inputs only. At two of the WwTWs (A and D), iron dosing (FeCl_3_) was being undertaken to reduce phosphate in the effluent via chemical precipitation into the sludge.

The effluent complexation characteristics were also compared with those for organic matter derived from a natural source, as described in the study by Constantino et al. (2015). For this, a 10-mg/L solution standard was prepared from the SRFA reference material obtained from the International Humic Substances Society (IHSS). The properties of SRFA have been extensively characterised and are considered to be broadly representatives of organic matter with a natural origin.

### Sample handling

Effluent samples were collected in acid-washed 10-L high density polyethylene (HDPE) containers between November 2008 and February 2009, respectively. The samples were filtered through 0.45 μm cellulose nitrate membrane filters (Whatman, UK) on-site and stored in acid-washed 10-L HDPE containers. Samples were packed in ice until returning to the laboratory where they were stored at 4 °C in the dark.

### Reagents and materials

All chemicals, unless specified, were obtained from Sigma (Gillingham, UK). All water was obtained from a Millipore Milli-Q ultrapure water system (referred to as MQ hereafter). All glassware was soaked in 1% HNO_3_ made up in MQ water and rinsed with copious quantities of MQ water prior to use.

### Analytical techniques

Sample pH was determined using a SENTEK P11 pH probe (Sentek, UK). Copper was quantified by graphite furnace atomic absorption spectrophotometer (GFAAS) using a Zeeman 4100ZL (PerkinElmer, Beaconsfield, UK) with a limit of detection (LOD) of 0.5 μg/L. Zinc was quantified by flame atomic absorption spectrometry, with a limit of detection of 5 μg/L. Iron and calcium were quantified using flame atomic absorption spectrometry using an AAnalyst 100 (PerkinElmer). Instruments were operated in accordance with the manufacturer’s recommendations and calibrated within a linear range (*r* ≥0.99) using standards prepared from 1000 mg/L spectroscopic standards diluted in MQ with 2% Optima grade HNO_3_ (Fisher, UK). All samples were measured in triplicate with precision expressed as relative standard deviation of less than 5% for individual titration samples. No certified reference materials were available for sewage effluents, but standard additions on individual titration samples provided 100% (±10%) recovery against the deionised water calibration.

Total organic carbon (TOC), dissolved organic carbon (DOC) and inorganic carbon were measured using a Model 700 TOC Analyser (O.I. Corporation, USA) in accordance with the manufacturer’s recommendations and which provided a limit of detection of <0.005 mg-C/L. Samples were measured in triplicate. The reagents were sodium persulphate (Na_2_S_2_O_8_) (100 g/L) and a dilute (5%) phosphoric acid solution. A 5-mg/L carbon standard was prepared weekly using potassium hydrogen phthalate.

### Synthetic chelating agents

The aminopolycarboxylate chelating agents ethylenediaminetetratacetic acid (EDTA), nitrilotriacetic acid (NTA) and diethylenepentaacetic acid (DTPA) were quantified using the method of Laine and Matilainen (2005). Analysis was performed on a Series 200 HPLC system (PerkinElmer, Beaconsfield, UK) using a 20-μL loop. Analytes were separated using a 250 mm × 4.6 mm-Kromasil C18 column. The mobile phase consisted of 0.03 mol/L sodium acetate, 0.002 mol/L tetrabutylammonium bromide as the ion-pair and 5% methanol, adjusted to pH 3.15 using formic acid. Calibration standards were prepared from ferric chloride, EDTA, DTPA and NTA as 0.1, 0.5, 1.0 and 5.0-μM solutions. The limits of detection were 0.21 μM (63 μg/L), 0.21 μM (83 μg/L) and 0.77 μM (148 μg/L), respectively.

The analytical protocol for aminopolycarboxylates required these to be reacted with Fe^3+^, since aminopolycarboylate-Fe^3+^ complexes are highly stable and display strong UV absorbance characteristics, which forms the basis for their quantification. The high stability of the Fe^3+^ complexes also suggests that if initially present in this form, these will be unable to dissociate and complex other metal ions. Consequently, concentrations of aminopolycarboxylate chelating agent were also distinguished in terms of their labile and non-labile (inert) concentrations. The analytical protocol required 1 mL of 1-mg/L FeCl_3_ solution to be added to 9 mL of 0.45-μm filtered sample and left to stand overnight. This converted the entire aminopolycarboxylate concentration into Fe^3+^ form, which was used as the basis for quantifying the total aminopolycarboxylate concentrations. Each sample was therefore also analysed without the addition of FeCl_3_ (MQ water was added instead) which enabled a measure of the inert aminopolycarboxylate. The labile aminopolycarboxylate concentration was calculated as the difference between the FeCl_3_ spiked and unspiked samples. Samples were analysed in triplicate.

### Complexation capacity titration

The Chelex column method (Gardner and van Veen 2004; Bowles et al. 2006) was used to assess complexation characteristics (binding capacity and binding affinity). The Chelex column method entails passing a water sample through a column packed with Chelex resin within which free or weakly bound metal ions that dissociate from complexes upon contact with the resin are retained. Complexation characteristics were determined by adding metal to aliquots of a 0.45-μm filtered sample and evaluating the pre and post-column concentrations. Complexation capacity represents the concentration at which the available complexing ligands become saturated such that an increase in the pre-column concentration does not result in an increase in the post-column concentration. The binding affinity of the ligands to which the added metal becomes bound is determined from the rate at which the post-column concentration increases. Since weakly bound metal species are retained within the column, this method provides a conservative characterisation of complexation behaviour. Furthermore, since the flow rate through the column and contact time with the resin influences complexation, the characteristics determined using this method are considered to be operationally defined.

The column preparation methodology is described in Constantino et al. (2015), with a brief explanation provided here. Calcium-form Chelex columns were prepared in borosilicate glass columns (Bio-Rad, USA) 50 mm (height) by 7 mm (internal diameter) with Chelex-100 as the chelating resin (200-400 mesh, Bio-Rad, USA). One millilitre of wet sodium-form Chelex slurry (equivalent to 0.15-g dry weight of resin) was inserted into each column and plugged with glass wool. The Chelex resin was converted to calcium form by pumping at least 10 mL of 2 M calcium chloride through each column at a flow rate of approximately 20 mL/min using a 302S/RL Watson Marlow peristaltic pump. For each sample, flow through the column was set at 50 (±2) mL/min. The pH of the column was stabilised between samples by passing at least 30 mL of reagent grade 0.1 M 3-(N-morpholino) propanesulphonic acid (MOPS) solution, amended to the sample pH, through the column. At least 12 aliquots of the 200-mL filtered sample were spiked with a metal standard prepared as 50-mg/L solutions in dilute Optima grade nitric acid (1%) (Fisher, UK) using reagent grade commercially available metal salts (Fisher, UK). Sample aliquots were spiked with either copper in increments of 50 μg/L or with zinc in increments 10 μg/L in order of increasing concentration. The volume of applied resin has a capacity to retain in excess of 10 mg of the considered metals so there was good confidence that the spiked metal would not exceed the resin capacity.

The pH of each aliquot was stabilised by adding 1 mL of reagent grade 1-M MOPS solution, prepared at the sample pH. The spiked aliquots were equilibrated for 1 h to standardise the kinetic conditions and passed through two separate columns (i.e. duplicate measurements for each spiked sample). The DOC concentration was constant in each titration. At least 40 mL was collected from each aliquot before being passed through the columns and 50 mL from each column after. Pre- and post-column samples were preserved by adding 1 mL of Optima grade nitric acid. Columns were replaced after each titration. Metal concentrations were quantified using either flame or graphite furnace spectrometry. The pre- and post-column concentrations represent dissolved and non-labile copper concentrations, respectively. The labile concentration may be determined as the difference between the pre- and post-column concentrations.

### Data treatment and analysis

A non-linear receptor binding equation (Motulsky and Christopoulos 2004a) was fitted to the titration data to estimate the complexation characteristics:

$$ Y=\frac{B \max \cdot X}{K\mathrm{d}+ X} $$

In the equation, *Y* represents the post-column DOC normalised metal concentration (in molar units). The metal concentration was normalised (metal concentration divided by the DOC concentration) to allow comparison of samples with different amounts of organic matter. The value for *X* represents the pre-column metal concentration (also in molar units). The point at which the available complexing ligands become saturated (i.e. complexation capacity) is represented by *B*_max_ and *K*_d_ the dissociation constant. The value for *K*_d_ is also the reciprocal of the complex formation constant (*K*) that is commonly used to describe binding affinity.

In order to estimate values for *B*_max_ and *K*_d_ and the uncertainty associated with these values, the data from the duplicate Chelex columns for each titration series were pooled in advance of the analysis. Pooling the titration data ensures that the estimated values for *B*_max_ and *K*_d_ take into account the effect of differences between individual columns due to random variation in the measured values. The best fitting values for *B*_max_ and *K*_d_ were those which minimised the sum of squared residuals and were determined using a customised Excel spreadsheet titration analyser. A Monte Carlo method was used to estimate confidence interval limits for *B*_max_ and *K*_d_ (Motulsky and Christopoulos 2004b). The residuals resulting from the best fitting values for *B*_max_ and *K*_d_ were applied randomly (with replacement) to the measured values to create an alternate titration series from which new values for *B*_max_ and *K*_d_ were estimated. The confidence interval limits for *B*_max_ and *K*_d_ were therefore the 2.5%ile and 97.5%ile values determined from 1000 simulations (i.e. the 95% confidence interval range).

**Fig. 1:**
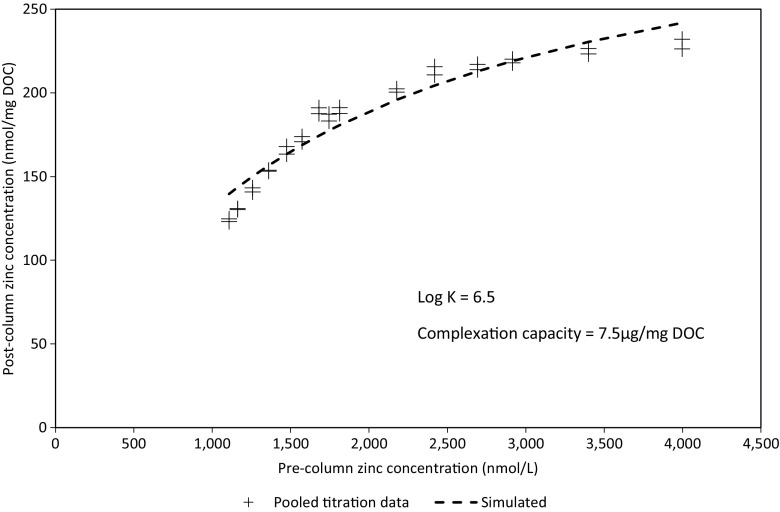
Chelex titration for zinc (sample B1)

An example of the titration data and fitted binding curve from which zinc complexation characteristics are derived (sample B1) is provided in Fig. 1.

The Chelex method and data analysis protocol is advantageous in that it enables a relatively rapid assessment of differences between samples and will clearly identify where the differences in complexation behaviour are significant. In reality, however, the ligands present in effluent and river waters represent a heterogeneous mixture of weak and strong binding sites and, consequently, representing complexation behaviour in terms of a single stability constant value will limit the extent to which the distribution of different categories of ligand may be discerned.

The differences in complexation characteristics between the various samples were evaluated using the corrected Akaike’s Information Criterion (AICc) approach which combines concepts from maximum likelihood theory, information theory and of the entropy of information (Akaike 1974; Burnham and Anderson 2002). A particular benefit of the AICc approach, and fundamental to its selection for this study, is that it enables a quantification of the magnitude of differences between samples rather than only whether differences are statistically significant. Using the AICc approach, a score is calculated for the complexation characteristics (*B*_max_ and *K*_d_) of each sample using the formula:

$$ \mathrm{AICc}= N\cdot \ln \left(\frac{\mathrm{SS}}{N}\right)+2 K+\frac{2 K\left( K+1\right)}{N- K-1} $$

The AICc score takes into account the number of parameters used to fit the complexation characteristics (*K*), the number of data points available in each titration (*N*) and the sum of squared residuals (SS). The differences between samples were evaluated through pairwise comparison whereby the titration data of paired samples (e.g. A1 and B2) are pooled and new complexation characteristics (e.g. A1B2) and AICc score determined. The difference between the AICc scores for the individual samples (e.g. A1 and B2) and the pooled sample (A1B2) are expressed as an evidence ratio (ER) and used to quantify the magnitude of the differences in complexation characteristics between samples. The ER is a likelihood value which, in the context of the present study, represents a measure of the difference in the complexation characteristics between the samples. Low ER values would indicate that a generalised set of complexation characteristics could be used to estimate bioavailability downstream of a discharge, with high ER values suggesting the opposite. ER values were calculated using the formula:

$$ \mathrm{ER}=\frac{1}{e^{-0.5\cdot \Delta {\mathrm{AIC}}_{\mathrm{C}}}} $$

## Results

### Effluent physico-chemical characteristics

The water chemistry characteristics are summarised in Table 1. The pH and DOC concentrations of all the sampled effluents were broadly similar (7.6 to 8.1 and 7 to 9 mg/L, respectively) and within the range expected for these effluents and wastewater treatment type (Gardner et al. 2012). The high calcium concentrations were indicative of a relatively hard water and were also within the range expected for waters within the region. The concentrations of iron were greatest in effluents B1 and B2, which were not dosed with iron to reduce phosphate in the final effluent. This reflected the influence of local geological characteristics but also the fact that because coagulant material had not been added to the wastewater, more iron would be retained in the aqueous phase. Of the synthetic chelating agents determined, only EDTA was detected and was found in all the effluents at significant concentrations (41 to 547 μg/L). NTA and DTPA were all below their limit of detection of 148 and 83 μg/L, respectively, in all samples. EDTA concentrations were greatest in the treatment works which received inputs from dairies (A and B, with 164 and 547 μg/L, respectively). The EDTA in the effluents from works which were treated with iron (A and D) was found to exist predominately (>79%) in the highly stable Fe^3+^-EDTA complex form (non-labile). Although the concentrations of iron were greatest in effluents B1 and B2, these effluents contained a substantial excess of labile EDTA, indicating that the iron in effluents B1 and B2 was not in free Fe^3+^ form (Table 1). The concentrations of copper and zinc in the effluents were within the range expected for the WwTWs reported in the study by Gardner et al. (2012), with the 5th, 50th and 95th percentile concentrations of dissolved Cu of 1.7, 5.6 and 19 μg/L, respectively, and the 5th, 50th and 95th percentile concentrations of dissolved Zn of 9.9, 24 and 59 μg/L, respectively.

**Table 1:** Effluent chemistry characteristics

Effluent sample	pH	DOC (mg/L)	Ca (mg/L)	Cu (μg/L)	Zn (μg/L)	Ni (μg/L)	Fe (μg/L)	EDTA^2^ (μg/L)
A1	7.6	7.5 (±0.1)	52 (±1)	2 (±0)	43 (±3)	5 (±1)	87 (±19)	547(±98) [0]
A2	7.5	7.0 (±0.1)	54 (±1)	5 (±0)	34 (±2)	10 (±1)	98 (±14)	393 (±76) [0]
B1	8.0	9.0 (±0.2)	69 (±1)	3 (±0)	68 (±1)	<2	131 (±30)	234 (±29) [120]
B2	8.1	8.2 (±0.1)	71 (±1)	2 (±0)	60 (±2)	<2	109 (±36)	164 (±10) [81]
C1	7.9	7.9 (±0.1)	54 (±1)	3 (±0)	46 (±6)	6 (±2)	26 (±24)	47 (±4) [0]
C2	7.9	7.9 (±0.1)	52 (±1)	4 (±0)	87 (±5)	8 (±1)	69 (±23)	41 (±3) [16]
D1	7.9	7.9 (±0.1)	40 (±1)	4 (±0)	57 (±2)	<2	36 (±5)	67 (±2) [0]
D2	7.9	8.3 (±0.1)	44 (±1)	3 (±0)	34 (±2)	<2	53 (±16)	124 (±8) [0]
UK median^1^	7.5	9.4	79	5.6	24	4.3	59	128

^1^Gardner et al. (2012). Mean concentrations for 162-UK WwTWs

^2^Numbers between brackets indicate 95% confidence interval range values. The values in square brackets indicate the labile EDTA concentration (i.e. not complexed with Fe^3+^)

### Copper complexation

**Fig. 2:**
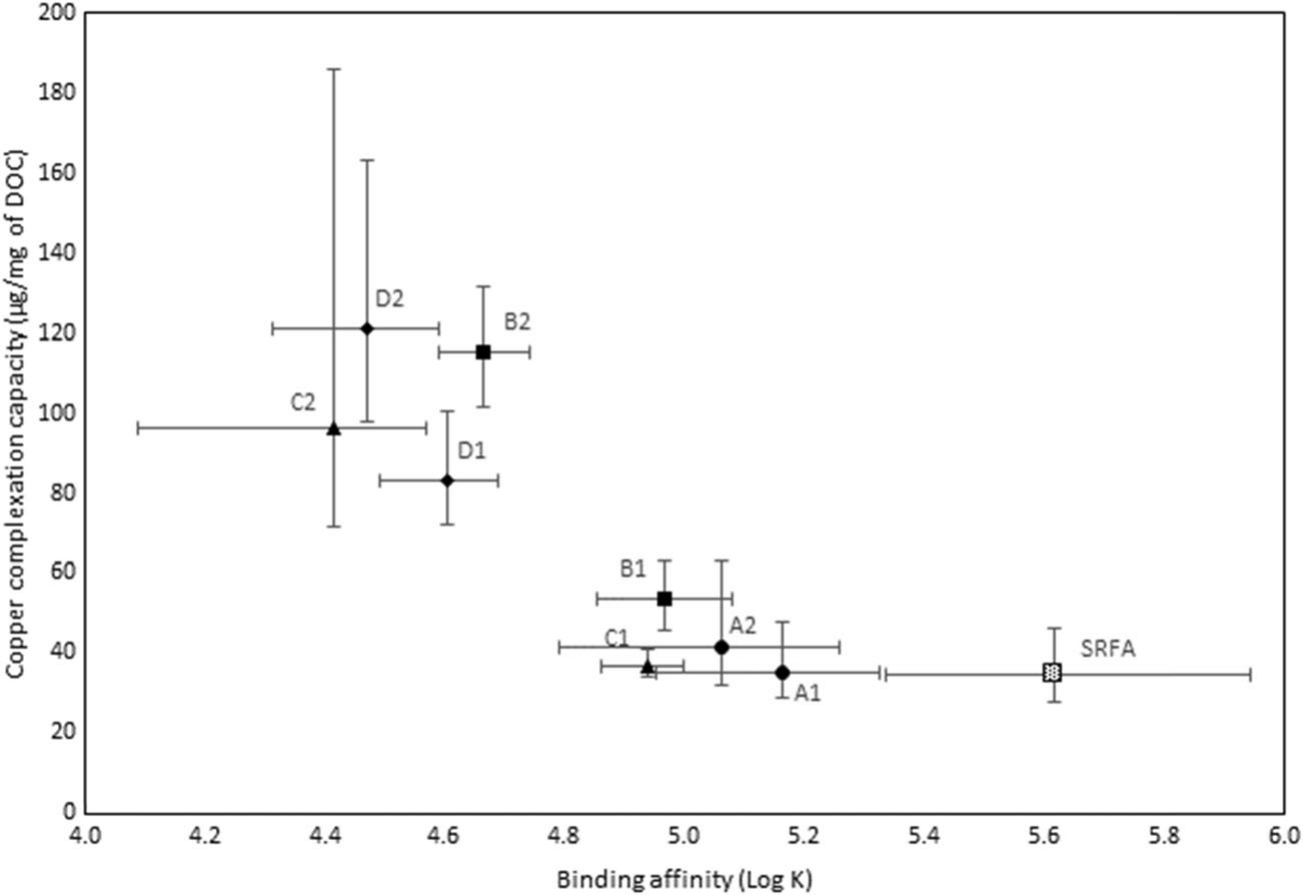
Copper complexation characteristics of sewage effluent (A1 to D2) and the Suwannee River fulvic acid

The copper complexation characteristics for the samples are given in Fig. 2. The data show that the DOC normalised complexation capacity for the effluents typically ranged between a factor of two of one another and exceeded that of SRFA by a factor of two on average.

The differences in complexation capacity were not correlated with concentrations of EDTA suggesting the differences to be attributable to other factors. The conditional stability constant values (log *K*) showed that the average binding affinity of the effluent-derived ligands was also lower than that for SRFA, indicating that on average, the effluents contained substantial concentrations of a category of moderately strong ligand (i.e. distinguishable by the Chelex method) but which were, nonetheless, weaker, on average, than the ligands detected in river waters. It was notable that the effluents with the higher complexation capacity values also demonstrated lower binding affinity values (log *K*), suggesting that the higher complexation capacity values are attributable to a greater concentration of weaker ligands. In all instances, complexation capacity was in vast excess (>factors of 10) of the copper concentrations typically found in effluent of sewage treatment works that receive inputs from predominantly domestic sources.

The AICc evidence ratios (Table 2) show that differences in the complexation characteristics of effluents from the same treatment works (e.g. A1 and A2) were generally smaller than those between different treatment works (e.g. A1 and C1). An exception was for samples B1 and B2, where B1 showed a smaller difference to D1 and D2 in comparison with B2. The data also show that differences between different effluents were generally smaller than the differences between the effluents and SRFA indicating that the complexation characteristics of effluents were more similar to one another than in comparison with SRFA. The implication is that effluent-derived ligands might indeed be realistically considered for inclusion as a distinct ligand category within the BLM. It is notable, however, that there were examples of effluents that were substantially more distinct from one another than from the SRFA sample. For example, the copper complexation characteristics for sample B2 were found to be more different from those of the other effluents than in relation to SRFA. Whereas B2 was found to have a substantial concentration of labile EDTA (80 μg/L), this was less than what was found in sample B1. The specific relevance of labile EDTA in respect to these differences could not, however, be directly ascertained.

**Table 2:** Akaike information criterion evidence ratios demonstrating the magnitude of the differences in copper complexation characteristics between samples

Sample	A1	A2	B1	B2	C1	C2	D1	D2	SRFA
A1		1	3	21,949	122	21	3	625	51
A2	1		2	727	186	21	2	4	30
B1	3	2		564	3336	224	11	2	20
B2	21,949	727	564		887,036	44,935	10,093	1320	40
C1	122	186	3336	887,036		15	2129	13,976	3361
C2	21	21	224	44,935	15		21	8	344
D1	3	2	11	10,093	2129	21		1	3932
D2	625	4	2	1320	13,976	8	1		65
SRFA	51	30	20	40	3361	344	3932	65	

Evidence ratios represent a relative likelihood value which quantifies the difference in the complexation characteristics between the samples. Lower numbers indicate smaller differences between samples and vice versa

### Zinc complexation

**Fig. 3:**
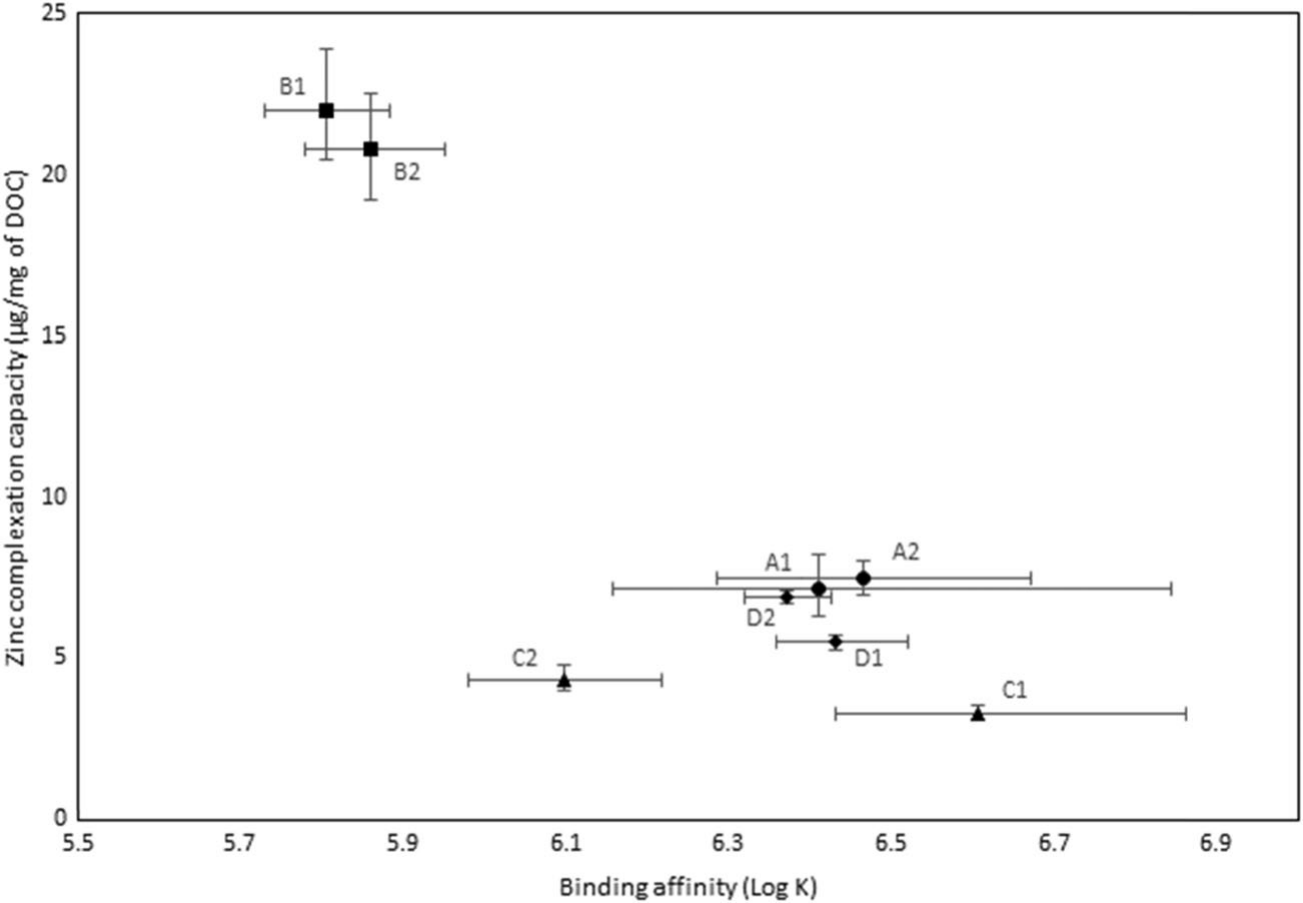
Zinc complexation characteristics of sewage effluent (A1 to D2)

The zinc complexation characteristics for the samples are shown in Fig. 3. For the effluent samples, the average concentration of zinc complexing ligands (per milligram of DOC) varied by a factor of three. Substantial differences in complexation capacity for samples B1 and B2, which were much higher in comparison with the other effluent samples were evident. Samples B1 and B2 also contained the highest concentrations of labile EDTA although the increased complexation capacity could not be directly attributed to the EDTA. As reported in Constantino et al. (2015), complexation characteristics could not be determined for the SRFA since the post-column concentrations of zinc were below the analytical limit of detection (<5 μg/L). The absence of post-column zinc indicates the binding affinity of the ligands present in the SRFA sample to be lower than the affinity between the Chelex and zinc and is in itself an indication of a substantial difference in complexation characteristics of organic matter derived from effluent and from natural sources.

The low affinity between zinc and organic matter derived from natural sources, evidenced by the inability to estimate complexation characteristics for SRFA using the Chelex method, however, suggests that the enhanced complexation capacity for B1 and B2 is likely to be attributable to synthetic or anthropogenically derived ligands. This also suggests that this ligand category is present at much higher concentrations in B1 and B2 in comparison with other effluents. Further detailed analysis of the DOC characteristics of the wastewater at this WwTW would be required to fully elucidate this data, for example assessing the impacts that molecular size distribution has on the Zn speciation (Chaminda et al. 2008) as well as determining the zinc forms such as the free metal ion concentration (Pearson et al. 2016).

The difference in the complexation profiles of the effluent samples and SRFA suggests that the effluents contained a category of ligand with an affinity for zinc that is substantially greater than those of ligands derived from natural sources. The implication is that effluent-derived organic matter may be more effective in mitigating the effects of exposure to zinc in comparison with organic matter derived from natural sources and consequently, that without consideration for these ligands, BLMs may overestimate zinc bioavailability in waters that receive significant effluent inputs.

The substantial differences in complexation characteristics between effluents are also evidenced by the AICc evidence ratios given in Table 3. The data show, as for copper, that the complexation characteristics of effluents from the same treatment works were generally more similar to one another (i.e. a smaller evidence ratio) in comparison with those effluents from other treatment works, excepting D1 which showed more similarity with A1 and A2 in comparison to D2. The AICc scores showed that the characteristics of A1 and A2 were least dissimilar to the other effluents and that B1 and B2 were most dissimilar. It is also notable that the ER values for zinc were substantially greater than the equivalent values for copper, demonstrating that zinc complexation characteristics were more variable than for copper. These data serve to illustrate that the challenge presented by the consideration of effluent-derived ligands within the BLM approach is that, in addition to the clear difference between effluents and naturally derived complexing ligands as seen for copper, there are also substantial differences in these characteristics between effluents.

**Table 3:** Akaike information criterion evidence ratios demonstrating the relative magnitude of the differences in zinc complexation characteristics between samples

Sample	A1	A2	B1	B2	C1	C2	D1	D2	SRFA
A1		1	5963	7328	4997	6182	187	10	nd
A2	1		24,870	30,656	37,896	35,488	1334	29	nd
B1	5963	24,870		1	5,192,022	4,687,719	1,543,302	1,078,170	nd
B2	7328	30,656	1		6,267,272	5,341,746	1,680,599	1,304,641	nd
C1	4997	37,896	5,192,022	6,267,272		2	14,078	247,367	nd
C2	6182	35,488	4,687,719	5,341,746	2		13,747	242,682	nd
D1	187	1334	1,543,302	1,680,599	14,078	13,747		1411	nd
D2	10	29	1,078,170	1,304,641	247,367	242,682	1411		nd
SRFA	nd	nd	nd	nd	nd	nd	nd	nd	

Evidence ratios represent a relative likelihood value which quantifies the difference in the complexation characteristics between the samples. Lower numbers indicate smaller differences between samples and vice versa

*nd* not detected/below limit of detection

## Discussion

The data presented here shows that the complexation characteristics of the effluent-derived organic matter offer a substantially greater (DOC normalised) capacity to complex copper and zinc than those derived from natural sources, which was also consistent with findings from other similar studies (van Veen et al. 2002; Sarathy and Allen 2005; Chaminda 2013). In their study, Sarathy and Allen (2005) proposed that up to 90% of the difference between river water and effluent copper complexation might be attributable to the influence of sulphide for which unprotonated sulphide clusters have been observed by others to form highly stable copper complexes (Rozan et al. 2000) (thermodynamic stability constant log *β* >54); however, since the average binding affinity values for the effluents in the present study were lower than the values for the SRFA sample, the additional complexation capacity observed for the sewage effluents is unlikely to have been substantially attributable to such a strongly binding ligand.

Sarathy and Allen (2005) also recognised that other complexing ligands were likely to occur in sewage effluent and that these were likely to be significant to copper speciation although they did not speculate on the identity of these ligands other than to suggest that these might be non-humic biological macromolecules. This suggestion is supported by the findings from a number of other studies which have investigated the chemical composition of sewage effluents (Ma et al. 2001; Pernet-coudrier et al. 2008) which found that approximately 50% of the carbon content in sewage effluent-derived OM was comprised of an acid soluble hydrophilic fraction made up of high molecular weight (>3500 Da) polysaccharides, in excess of the proportion which occurs in naturally derived organic matter (between 9 and 30%). In their study, Ma et al. (2001) also investigated the copper binding characteristics of the hydrophilic fraction and found this to have an affinity for copper which was lower than that between copper ions and humic or fulvic acids. Chaminda et al. (2008) investigated Zn and Cu complexation in WwTW effluents and showed over 75% of the metal present bound, with most of the copper associated with a <500 Da fraction, with Zn exhibiting a more variable distribution. Labile species were also found within the larger fractions confirming that they were not present as the free metal ion. Other components of sewage, although not extensively researched, are also likely to have an impact on copper and zinc complexation, such as amines, although their strength of binding is relatively considered weak (Manceau and Matynia 2010). This provides a plausible explanation for the finding in the present study that the binding affinity of the effluent-derived ligands was generally lower than the binding affinity of the ligands in the river samples, which, consequently, implies that a substantial proportion of the additional complexation capacity observed in the present study may indeed have been attributable to high molecular weight non-humic substances.

Furthermore, what is immediately evident regarding the binding strength of the effluent ligands is that conversely to that observed for natural ligands, the stability constants are actually higher for zinc than for copper. The data presented here with mean log *K* values ranging from 5.5 to 6.6 are in line with that published previously where a two ligand complexation capacity system was assumed with log *K* values for three WwTW ranging from 6.2 to 7.4 (compared with copper log *K* values of 6.4 to 7.7), suggesting there are ligands present in effluents which have at least as strong or even a stronger affinity for zinc than for copper (Chaminda et al. 2008). Since the complexation characteristics derived via the Chelex method are operationally defined, and that binding behaviour is described using a single stability constant value, it is plausible that the stability constant for copper includes the effect of a greater number of relatively weak ligands and for zinc a smaller number of relatively strong ligands. This is supported by the fact that the studies by Ma et al. (2001) and Pernet-coudrier et al. (2008) did not consider the potential significance of anthropogenic ligands, such as EDTA, which is known to have a high affinity for copper and zinc and is expected to occur in sewage effluents (Knepper 2003; Knepper et al. 2005). Indeed, a study Baken et al. (2011), which investigated the metal complexing properties of organic matters in anthropogenically impacted surface waters, identified the significance of synthetic chelating agents such as EDTA to trace metal speciation.

In their Chelex method development, Bowles et al. (2006) used EDTA as a model complex to provide an indication of the Chelex method’s ability to discriminate between ligands of different strengths. Their study found that >95% of Cu and Zn EDTA complexes were non-labile (i.e. passed through the Chelex column). However, their study did not consider the original form of the EDTA and this is likely to be important. In the present study, the labile EDTA might plausibly contribute a complexation capacity of 2.9 μg/mg of DOC for copper in sample B1 (the highest labile EDTA concentration, assuming 1:1 stoichiometry) versus the total complexation capacity of 53 μg/mg of DOC (c5% of the total). Similarly, for zinc, the labile EDTA could plausibly explain complexation capacity of 3.0 μg/mg of DOC in sample B1 versus the total complexation capacity of 22 μg/mg of DOC (c14% of the total). Whereas EDTA is perhaps the most well-known synthetic chelating agent, numerous other anthropogenic substances with chelating properties are also known, or may reasonably be expected, to occur in sewage effluents. In the context of the present study, whereas it is plausible that some proportion of the difference between the complexation capacity values for the effluent and SRFA might also have been attributable to sulphide, it is perhaps more plausible that this was attributable to the presence of biological macromolecules, as well as to synthetic chelating agents and/or their breakdown products.

The determination of metal speciation in complex matrices such as wastewaters is not straightforward and a number of operationally defined assumptions are applied, particularly, when setting competition strengths to determine detection windows for relevant ligands. In this case, a relatively simple Chelex-based method has been used with a single conditional complexation reaction set up. Other authors have implied that two ligand system (Chaminda et al. 2008) or various models may be used to describe the observed complexation (Lofts and Tipping 2011). The availability of binding sites at defined ligand strengths will be a function of the concentration of metal added as part of the titration process, the metal present in the sample, and the binding sites present (Town and Filella 2000). In reality, there is a wide spectrum of ligands present in effluent and aquatic environments across many orders of magnitude strength and complexation capacity.

Consequently, conclusions regarding the ligand complexation in its broadest sense, particularly when comparing data between different studies, can only provide indicative rather than absolute conclusions. However, when doing relative comparisons such as those provided in the present study, conclusions regarding relative differences between samples can be drawn with confidence.

The findings from this study therefore contribute support to the view that the influence of effluent-derived organic matter should be taken into account within the context of biotic ligand modelling but that further research is required to better understand the reasons for differences in complexation characteristics between effluents. This is of particular significance for zinc for which the differences in complexation characteristics for organic matter derived from effluent and those from natural samples were greatest.

## Conclusions

This study has showed that sewage effluents contain a category of ligand that display complexing characteristics that are different from ligands present in samples derived from natural sources. These differences are substantially greater for zinc than those for copper. The copper and zinc complexation characteristics of effluent samples obtained from the same treatment works were less different from one another than in comparison to effluents from other treatment works. Since all the sewage treatment works utilised the same treatment technology, this finding suggests that sewage source has an important influence on complexation characteristics. Zinc complexation characteristics of the effluents were substantially different to those for the river-derived fulvic-acid-tested sample. The complexation capacity of effluent exceeded that available in natural samples by up to a factor of at least 4, suggesting that current biotic ligand models might overstate the risk of harm from zinc in effluent-impacted waters. The implication is that by ignoring the effect of effluent-derived ligands, the current approach is likely to provide a conservative assessment of risk. The fact that differences in complexation characteristics between different effluents was greater for zinc than for copper suggested that a generalised set of complexation characteristics may be more challenging to define. EDTA, a common complexing agent used in domestic and commercial products, was detected in all the effluents although not all of it in the labile form. EDTA is likely to have contributed to the differences in complexation capacities observed for zinc and will be more significant for metals such as zinc which has a low affinity for naturally derived ligands. The findings from this study support the case that the contribution to complexation from effluent-derived ligands will enhance the environmental relevance of bioavailability-based compliance criteria, for zinc in particular, and should therefore be considered for the purposes of compliance-based biotic ligand modelling.
